# Origin of Angiosperms: Problems, Challenges, and Solutions

**DOI:** 10.3390/life13102029

**Published:** 2023-10-09

**Authors:** Xin Wang

**Affiliations:** State Key Laboratory of Palaeobiology and Stratigraphy, Nanjing Institute of Geology and Palaeontology and CAS Center for Excellence in Life and Paleoenvironment, Chinese Academy of Sciences, 39 East Beijing Road, Nanjing 210008, China; xinwang@nigpas.ac.cn; Tel.: +86-25-8328-2266

**Keywords:** origin, angiosperms, misconcept, fossil, flower, hypothesis, Unifying Theory

## Abstract

Angiosperms are frequently assumed to constitute a monophyletic group. Therefore, the origin of angiosperms is a key question in systematic botany since the answer to this question is hinged with many questions concerned with angiosperm evolution. Previously, the lack of fossil evidence undermines the robustness of related hypotheses, and explains the instability of the systematics of angiosperms in the past century. With increasing evidence of early angiosperms, the origin and early evolution of angiosperms become approachable targets. However, reaching a strict consensus is still a mission impossible now: there are too many issues open to debate. A good sign in research is that palaeobotanists started addressing the issue of criterion identifying angiosperms, this would bring order in studies of early angiosperms. Several flaws in fundamental concepts inflicting botany require efforts to elucidate and remedy. The author here opens a discussion on these problems, hoping that more botanists will join to discuss and clarify previously blurry concepts and place a solid foundation for future development in botany.

## 1. An Important but Perplexing Question

The term “angiosperm” was coined in 1690 by the German botanist Paul Hermann (1646–1695) from Greek *angeion* (vessel) + *spermos* (seed), to name those plants that have their seeds contained, in contrast to “gymnosperms” that have their seeds exposed. In the current world, there are over 300,000 species of angiosperms, accounting for over 90% of the plant species diversity [1]. This single group of plants provide most of the food and materials necessary for the sustainable development of humans. Without them, the occurrence of humans on Earth would be inconceivable. Needless to say, angiosperms are the single most important plant group for us. It is natural for many botanists to focus their attention on angiosperms and study various aspects of angiosperms. Among these questions concerned with angiosperms, a fundamental question in botany is the origin of angiosperms, namely, from which group the angiosperms were derived. Since angiosperms are frequently taken as a monophyletic group, how the earliest angiosperms looked is a key question in angiosperm systematics. This explains why palaeobotanists make efforts to seek fossils of the earliest angiosperms. Over centuries botanists (including palaeobotanists) have been painstakingly working to answer this question.

## 2. Conflicting Answers for the Same Question

A natural but unpleasant status of current studies is that, rather than reaching a consensus, several different and conflicting answers have been given to the same question. Since it is a mission impossible to give an exhaustive review of all these works in this short paper, I prefer to focus my review on questions of great influence in the past couple of centuries. 

“Angiosperm” is a well-defined botanical term. From the first day when Paul Herman (1646–1695) coined the term “angiosperm” in 1690, the meaning of the term “angiosperm” has been clear. Per the definition of the term angiosperm, angiosperms are distinguished from gymnosperms by their reproductive parts (flowers) that contain their seeds/ovules, which is not seen in gymnosperms. Therefore, the core question in the origin of angiosperms is how previously naked seeds/ovules became enclosed in the ancestral angiosperms that may have little morphological difference from their gymnosperm peers. As for the provenance of angiosperm flowers, there used to be a confrontation between the Pseudanthial Theory and the Euanthial Theory (also known as the Anthostrobilus Theory). The Euanthial Theory thought that angiosperms were derived from Bennettitales-related groups, unisexual flowers were derived from former bisporangiate strobili, and apocarpous Magnoliales were the most ancestral group among extant angiosperms [2]. While the Pseudanthial Theory thought that angiosperms were derived from Gnetales, and amentiferous-hamamelid orders Casuarinales, Fagales, Myricales, Juglandales, Chloranthaceae, and Piperaceae represented the most ancestral groups in extant angiosperms [3]. These two schools were stalemated around 1900, and the balance between them was historically broken by a slight impetus: the occurrence of fossil evidence of bisexual reproductive organs in Bennettitales [4,5,6]. Although current morphological knowledge of Bennettitales has rejected comparability and phylogenetic relationship between Bennettitales and Magnoliales, bisexuality and spatial deployment of male and female parts in reproductive organs in both groups did break the delicate balance between two confronting schools: Arber and Parkin [2] declared the victory of Euanthial Theory over Pseudanthial Theory. Thereafter Pseudanthial Theory permanently lost its voice in botany, although both theories have now been discarded due to a lack of favoring fossil evidence and emerging molecular systematics. The current mainstream angiosperm system (APG system), however, was more or less born out of and at least initially calibrated by the Euanthial Theory.

Angiosperms have various ways to enclose ovules in their gynoecia. Since the basic and simplest unit of an angiosperm gynoecium is termed “carpel”, the nature of carpels naturally is a focus of frequent studies and debates in botany. There used to be two confronting schools on this topic, namely, phyllosporous and stachyosporous schools. The phyllosporous school stated that a carpel was equivalent to a “megasporophyll” ( a leaf) bearing ovules along its margins. This idea has been popular in botany more or less due to the celebrity Johann Wolfgang von Goethe (1749–1832), who was famous in botany for his dictum “Alles ist Blatt” (“all is leaf”). This school used to dominate botany for a long time, and carpels in Magnoliales were assumed to be derived through longitudinal enrolling of the assumed megasporophylls in the Euanthial Theory [2]. On the contrary, the stachyosporous school stated that ovules were borne on a branch/branches [7]. The examples favoring this school include Centrosperms, which frequently have free central placentation [8]. 

## 3. Reasons Underlying the Controversies

In the debates on the nature of carpels, botanists in both schools have been working hard to convince the other with their own evidence, but neither succeeded. Why? Because both schools have found and shown evidence favoring their own ideas in extant plants that are all from the same time plane. The aims of these scholars were to reconstruct the deep history before extant plants, but the great diversity of extant angiosperms does not disfavor any hypotheses, just as the infinite number of straight lines go across a single point. Without time information embodied only in fossils, cherry-picking favoring evidence among diverse gynoecia and carpels in extant angiosperms is an easy job and allows any saying to go. Focusing on a favoring feature of selected groups allows one easily to be blind to unpleasant evidence and alternatives from the others. With valuable time information that is lacking in extant plants, plant fossils provide crucial independent data testing and sifting a valid hypothesis out of many proposed candidates. 

## 4. False Evidence Favoring the Euanthial Theory

The dominance of the Euanthial Theory is not a random aftermath. Many publications favored this theory, and these publications (especially those by famous authors) persuaded many otherwise hesitant botanists and consolidated the foundation for the theory. The Euanthial Theory started its dominance in the war against the Pseudanthial Theory when Arber and Parkin published their paper titled “*On the origin of angiosperms*” in 1907, taking advantage of then just published bisporangiate strobili of Bennettitales [6]. The bisexuality and proximal arrangement of androecium and gynoecium in Bennettitales were compared to those in Magnoliales and interpreted as evidence favoring the Euanthial Theory. This conclusion was further repeatedly strengthened by several ensuing publications of influence published in the past century, including anatomic work of Canright [9], studies of fossil early angiosperm *Archaeanthus* by Dilcher and Crane [10], *Archaefructus* by Sun et al. [11], and *Monetianthus* by Friis et al. [12]). These works initially were interpreted as favoring the Euanthial Theory. However, more detailed examinations indicate that these favorings are all spurious (see below).

## 5. Emerging Inconsistencies and Lacunae in Proof

If all evidence favored the Euanthial Theory, then any new angiosperm system, including the APG system, would not occur. As time passed, increasing lacunae in the proof of Euanthial Theory popped up. The earliest and alarming one is from Parkin (one of the only two authors of Arber and Parkin [2]) himself: Parkin admitted in 1925 (Arber passed away in 1918 [13]) that their conclusion made eighteen years before had no fossil evidence support [14]. In the fossil record, at least as early as 1984 some negative evidence started to emerge. Friis [15] reported over 100 angiosperm fossil taxa from the Late Cretaceous of Sweden. It is noteworthy that, despite the abundant and diverse taxa reported in the fossil flora, no trace of Magnoliaceae was found. This lack of Magnoliaceae in the fossil flora was quite conspicuous and alarming, especially when the Euanthial Theory was still of great influence. However, both of the above important alarming signals were either downplayed or largely ignored, (intentionally?) successfully. 

One cannot help asking, “Are these publications peerless, singular exceptional exceptions? Should they be reasonably ignored or downplayed, considering more other publications favoring the Euanthial Theory?” Reviewing these “publications favoring the Euanthial Theory” indicates that at least some of the other favoring-appearing publications have been involved in various forms of data misinterpretation or even falsification, and their corresponding supposed favor for the Euanthial Theory should either be discarded, discounted, or at least re-evaluated. 

According to the Euanthial Theory, the ovules in Magnoliaceae should be borne on the ventral margin of carpels, as ovules are supposed to be borne on the margins of megasporophylls. This expectation was met by Canright using his anatomic work: Canright [9] provided some specious anatomic evidence favoring the theory, based on his anatomic study of magnoliaceous fruits/carpels. Indeed, he appeared to have succeeded, as no one (at least the reviewers of his paper did not) realized that Canright had altered the data about the connection of ovule-bearing vascular bundle to meet the theoretical expectation of the Euanthial Theory. In the only picture demonstrating ovule-bearing vascular bundle provenance in *Magnolia salicifolia* Maxim. (his Figure 8), the ovule was connected to the “cort. b.” (cortical bundle), but the same ovule was drawn to be connected to the “v. b.” (ventral bundle) in his Figure 17 [9]. It appeared that his subtle change escaped the attention of the reviewers, who probably also expected the situation in his Figure 17. This case is not peerless. Instead similar case also occurred in some studies of fossil angiosperms: Dilcher and Crane [10] drew ovules of *Archaeanthus* on the ventral margins of carpels in their Figure 60h (just as the Euanthial Theory expected), their own Figure 24 indicated that at least some of the ovules were borne on the dorsal fruit margin [10]. In addition, the famous early angiosperm *Archaefructus* was initially described as bearing “follicles”, implying that ovules were borne on the ventral margin of carpels in *Archaefructus* [11]. This “rational” implication congruous with the Euanthial Theory was later found groundless as the ovules were later found borne along the dorsal margin of the carpels/fruits [16]. In both of the above two cases, how these “correct” and “rational” mistakes had escaped the supposed careful examinations of the reviewers is a mystery. A rational guestimation is that the presented information was compatible with pre-existing one, which has long been imbibed in the mind of reviewers beforehand. In addition, the almost universally expected ventral position of ovules in carpels has been disfavored by Endress’*Brasenia* ovule borne on the dorsal (rather than ventral) vascular bundle [17]. 

According to the Euanthial Theory, the primitive ovules in angiosperms should have two integuments (which is the status of ovules in all assumed basal angiosperms and is immune to changes in angiosperm system changes), just as believed and stated by Herendeen et al. [18]. Compatible with this expectation, Friis et al. [12] claimed that *Monetianthus* (a charcoalified fossil assigned to Nymphaeales by the authors) had two integuments and supported their claim using their Micro-CT virtual section (their Figure 5f). However, no one can see the second integument in their figure: there was only one layer of tissue that could be interpreted as an integument. The erroneousness of the interpretation by Friis et al. [12] became more obvious when their Figure 5f is put side by side with the supposed standard two integuments (Figure 2h of Herendeen et al. [18]) for comparison: even for someone who does not know how to count, the difference in number of integuments in these figures is obvious. It is noteworthy that Friis et al. cannot reject the “standard two integuments” proposed by Herendeen et al. [18], as Friis et al. and Herendeen et al. are almost the same group of authors (except Herendeen alone). Friis et al.’s [12] claim of two integuments in the ovule of their Cretaceous angiosperm, although theoretically expected, actually lent no support to the Euanthial Theory. Actually, having two integuments has nothing to do with being angiosperms or not, as *Cordaianthus duquesnensis*, a gymnosperm from the Palaeozoic, has two integuments (Figure 14 of [19]).

## 6. “Sporophyll” and the Euanthial Theory

“Sporophyll” is a frequently and widely used term for seed plants in botany textbooks. It has a far-reaching influence on almost all botanists. To many, reproductive organs of seed plants cannot be themselves without “sporophylls”. All sporangioids (sporangia, pollen sacs, and ovules) in ferns, gymnosperms, or angiosperms were interpreted as borne on “sporophylls” in currently available botany textbooks. “Sporophyll” is one of the few founding concepts underlying the Euanthial Theory. In the Euanthial Theory, a carpel was interpreted as a “sporophyll” bearing ovules along its margins and enclosing the ovules through enrolling along its longitudinal axis.

“Sporophyll” was so popular in botany for reasons. According to Arber and Parkin [2], they interpreted a carpel as derived from a leaf, at least partially, because of Goethe’s dictum “All is leaf”. Goethe (an amateur botanist [20]) is not the inventor of this idea. Far before Goethe, botanists Grew and Wolff had made similar statements in 1672 and 1768, respectively [21]. The influence of these botanists appeared little in botany. Celebrity and appealing expression won over professionalism, even among the assumed scientific professional botanical circle: Goethe had more heavily influenced Arber and Parkin, who won the debate against the Pseudanthial Theory and initiated the dominance of the Euanthial Theory while citing the dictum of celebrity Goethe [2].

**Figure 1:**
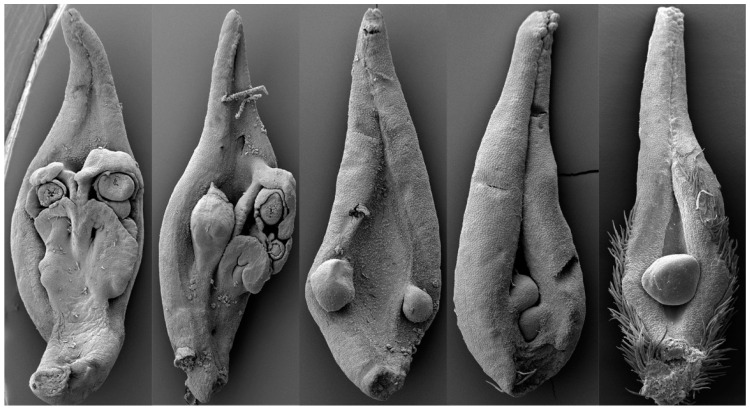
The variation spectrum of carpel morphology in a single tree of *Michelia figo* (Magnoliaceae) reveals that a carpel is a composite organ derived from the synorganization of a leaf and its axillary ovule-bearing branch (placenta). Courtesy of Zhang et al. [30].

The validity of Goethe’s statement was recently challenged by evidence from several lines. First, the most leaf-like female reproductive parts, “megasporophylls”, in all living plants, of *Cycas* were proven, by a developmental experiment, to be leaf-like due to mechanical pressure but not genes [22]. Second, vascular bundles in at least some cycad “megasporophylls” furcate in three dimensions, rather than in a single plane as expected for a typical leaf [23,24]. Third, in the earliest cycad fossil “megasporophyll”, of Permian *Primocycas* [25], the ovules were not borne along the supposed leaf margins. Fourth, the currently available fossil evidence [26,27] indicates that all sporangia in all early land plants are borne on the termini of branches rather than on any leaf margins. Since ovules and pollen sacs are all derived from these terminal sporangia, the supposed marginal position of ovules, if indeed so, must be a consequence of long-time evolution rather than an ancestral status in seed plants. Fifth, Crane has repeatedly stated that, at least in some Mesozoic fossil plants, the ovules are borne on the termini of branches rather than leaf margins [28,29]. Sixth, the carpels of *Michelia* (Magnoliaceae) have been proven synorganizations of a placenta and its subtending leaf (Figure 1), rather than metamorphs of leaves [30]. It appears that Goethe’s conception has misled almost all botanists over centuries [31], and it is time to clean it out of botany, at least that of seed plants.

The term “sporophyll” is a misconception that should be discarded in the botany of seed plants, despite its wide usage and popularity. The removal of “megasporophylls” undermines the validity of the Euanthial Theory, as the former is a core concept founding the latter.

## 7. Wrangles over the Criterion of Angiosperms

Despite the importance and great diversity of angiosperms and the initially clear definition of angiosperms, surprisingly, there was no general consensus on the criterion of angiosperms. The wrangling over the criterion of angiosperms has especially intensified in recent years. For example, since 2017, Herendeen et al. [18], Wang [8], and Bateman [32] have advanced their own different criteria for identifying angiosperms. Herendeen et al. [18] listed several features as “unique” of angiosperms (including enclosed ovules), but the inapplicability of their criterion was obvious: their exemplar fossil angiosperms published by themselves [10,12,33] did not have all the listed features, furthermore, their work published after 2017 [18] did not have these features, either. Bateman [32] proposed double fertilization and closed carpel as a criterion for angiosperms (a criterion distinct from that proposed by Herendeen et al. [34]) as if double fertilization were restricted to angiosperms. However, the long-existing botanical facts are that double fertilization has been seen not only in angiosperms but also in gymnosperms [35,36] and that there is no double fertilization in some angiosperms [37]. 

As early as 2002, Tomlinson and Takaso [38] found that the critical difference between gymnosperms and angiosperms is whether ovules are enclosed at the time of pollination. This criterion is an over-strict one in practice, as some angiosperms (for example, *Reseda*, [39]) may have their ovules exposed throughout their lives and thus would be placed in gymnosperms if this criterion were strictly applied. Despite this shortcoming, this criterion has its peerless advantage over others: Any plant meeting this criterion is unexceptionally an angiosperm. This explains why Wang [8] proposed “ovules enclosed before pollination” as a criterion for identifying fossil angiosperms. Among all the criteria proposed, this one has a meaning closest to that of the term “angiosperm” when it was coined by Paul Hermann more than 300 years ago.

It is noteworthy that the above-adopted criterion identifying angiosperms does not specify what encloses the ovules. The answer to this question is open, either a leaf, a branch, or their combination. This openness allows the diversity of early flowers and their bearers (early angiosperms), and explains why some of the early angiosperms including *Schmeissneria*, *Nanjinganthus*, *Qingganninginfructus*, and *Taiyuanostachya* have unexpected flower morphologies.

## 8. A Grade of Evolution

A general evolution trend in sexually reproduced organisms is termed “ODC” (offspring development conditioning) [40]. According to the theory of ODC [40], all sexually reproduced organisms (including plants and animals) are especially vulnerable to various harms in their early development stage, and whoever provides enhanced control over the developmental environment of its offspring has a better chance to succeed in its survival struggle. This generalization is applicable not only for extant angiosperms that include over 300,000 species, but also for fossil plants that are dated at least back to the beginning of the Devonian (>419 million years ago). The above-mentioned distinction between angiosperms and gymnosperms, ovule enclosing, is one of many implementations of ODC. However, even in angiosperms, the implementation of ODC is fulfilled in various ways. The great diversity of ovule-enclosing ways in angiosperms [41] implies that ovule-enclosing is one of multiple proxies of evolution grades that may be achieved multiple times independently, rather than a feature idiosyncratic of a single plant group that can be achieved at one time and in a single way.

This conclusion is closely hinged with the monophyly of angiosperms, which is currently well-accepted as a monophyletic group. New knowledge of angiosperms, fossil and extant, seems to suggest that the multiphyly of angiosperms cannot be excluded from the alternative list for the time being. If so, a fundamental change in the systematics of angiosperms will occur in the near future, as many related hypotheses are hinged with the monophyly of angiosperms and will need re-evaluations.

## 9. Resorting to the Fossils

**Figure 2:**
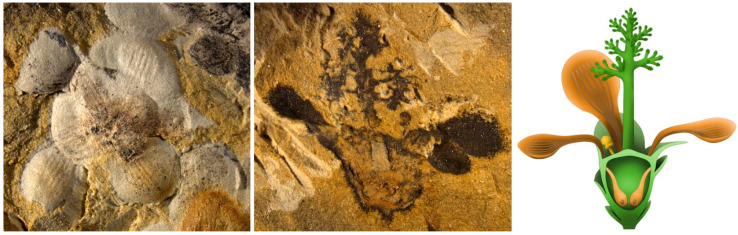
*Nanjinganthus dendrostyla*, a Jurassic angiosperm from China, with an inferior ovary and a dendroid style confirms the pre-Cretaceous existence of angiosperms. Courtesy of Fu et al. [42].

The origin and evolution of angiosperms deal with the vanished history of angiosperms. Since it has vanished, the history of angiosperms cannot be safely and correctly inferred and reconstructed based on information embodied in extant plants alone. The big but rarely asked question is “How can we test our extant-based conclusion scientifically?” Whether the gene evolutionary trends or regularities generalized from DNA sequences available only from extant angiosperm materials that cannot be older than a couple of hundred years are applicable to the million-year-long history of angiosperms is an open question, just as you may not predict the temperature of a day one year later using the temperature of yesterday and today alone as your all data. It is impossible for any of us to escape the limits of currently available technologies. Before we invent a time machine or find a wormhole, fossils are the only reliable information source for plant history, just as antiquity for human history. Any thought or theory about plant history, no matter who is/are the author(s), should be examined and tested carefully using fossil data. A good sign is that meaningful fossil evidence has started to emerge, for example, *Combina* from the Triassic of Spain gives new insight on the homology of carpels. Furthermore, the occurrence of inferior ovary in both *Nanjinganthus* from the Jurassic (Figure 2) [42] and *Monetianthus* from the Cretaceous [12], published by different authors, is unexpected by the traditional theories. However, if such ovaries are interpreted as a consequence of expansion and invagination of the flower axis, their early occurrence and lack of carpels are rather rational expectations. This possibility becomes more plausible when the following reason is taken into consideration: (1) expansion and invagination of the flower axis are so far never seen in any gymnosperms, and (2) expansion and invagination of the flower axis has been documented in the currently assumed basalmost angiosperm, *Amborella* [43].

## 10. Pre-Cretaceous History of Angiosperms

“How old are angiosperms?” Scientists have been trying hard to answer this question from various aspects, including fossil and molecular clocks [44]. The abundant specimens of *Schmeissneria* [45], *Nanjinganthus* in the Early Jurassic (Figure 2) [42], and *Qingganninginfructus* [46] refute the stereotype “No angiosperms until the Cretaceous”. Furthermore, recent new progress shone a spotlight on a new angiosperm from the Early Permian (>272 Ma), *Taiyuanostachya* [47]. Unlike other previously reported fossil angiosperms, both angiospermy and angio-ovuly (characteristics of angiosperms) have been demonstrated in *Taiyuanostachya*, making it an unequivocal angiosperm from the Palaeozoic. Current observation suggests that the seclusion of the ovule in *Taiyuanostachya* is implemented in a mode similar to that in basal angiosperms [48]: by secretion (not physical seclusion)! Considering that the mainstream idea about angiosperm history is still that angiosperms did not occur until the Cretaceous [18,49], many misled leading botanists should update their image of angiosperm evolution with the latest progress of palaeobotany.

## 11. Reconciling the Stalemate Confrontation

One may wonder, “Which of the above opposite parties, for example, phyllosporous and stachyosporous schools, is wrong or right? How did they stalemate, considering there was so much testing data available from extant angiosperms? Is there any way to reconcile them?” Fortunately, there seems to be a plausible and acceptable solution for reconciling these two confronting schools. Unlike the previous botanists insisting either on the foliar nature or axial nature of carpels exclusively, Wang [8] proposed, based on studies of fossil as well as extant angiosperms, that a carpel is a composite organ composed of a foliar part (ovary wall) and an axial part (placenta = ovule-bearing branch). This proposal is not completely new, as decades ago various authors had made more or less similar proposals, trying to derive carpels from Gnetales, Ophioglossaceae, or Glossopteridales [50,51,52,53,54,55,56]. However, the newer version of this proposal is in line with the recent treatment of a carpel as a bipartite organ resulting from the synorganization of ovules and carpel (wall) [57]. Reviewing previous works indicates that the opposite phyllosporous school and stachyosporous school were correct in their own aspects: both schools focused on two different-natured carpel parts, they all were honest and emphasized their preferred aspects of the same truth about carpels, which include both foliar and axial parts, and, unfortunately, both of them refused to compromise and accept the other’s evidence. If they had integrated the evidence from the opponents into their consideration, they should have long come up with a more comprehensive and win-win point of view on the nature of carpels. The long-lasting confrontation between phyllosporous and stachyosporous schools disappears when a carpel is taken as a composite organ including both foliar and axial parts.

## 12. End of the Evolution Story?

In the currently available botany textbook, angiospermy and angio-ovuly appear to culminate in the evolution of seed plants. However, they are not and cannot be the end of plant evolution. According to the theory of ODC [40], land plants (including extant angiosperms) will not conclude their evolution now, instead, they will continue to enhance their ODC in various ways and thus extend their phylogeny. More and further ways (other than angiospermy and angio-ovuly) extending plant evolution have occurred and have been exemplified by some angiosperms, for example, Laurales (Calycanthaceae, Monimiaceae, Siparunaceae) [58,59,60] and Moraceae [59] have developed various forms of hypanthia that enclose and protect their carpels/fruits (angiocarpy), and Rhizophoraceae have developed vivipary as a strategy to ensure nutritional supply for their offspring [61]. Apparently, the evolution story will continue and plants will flourish in their ways.

## Data Availability

Data sharing not applicable.
